# The transcriptome landscape of *Prochlorococcus* MED4 and the factors for stabilizing the core genome

**DOI:** 10.1186/1471-2180-14-11

**Published:** 2014-01-18

**Authors:** Bang Wang, Lina Lu, Hexin Lv, Huifeng Jiang, Ge Qu, Chaoguang Tian, Yanhe Ma

**Affiliations:** 1 Key Laboratory of Systems Microbial Biotechnology, Tianjin Institute of Industrial Biotechnology, Chinese Academy of Sciences, Tianjin 300308, China; 2 Institute of Microbiology, Chinese Academy of Sciences, Beijing 100101, China

**Keywords:** Core genome, Gene expression, Molecular evolution, *Prochlorococcus*, RNA-Seq, Transcriptome

## Abstract

**Background:**

Gene gain and loss frequently occurs in the cyanobacterium *Prochlorococcus*, a phototroph that numerically dominates tropical and subtropical open oceans. However, little is known about the stabilization of its core genome, which contains approximately 1250 genes, in the context of genome streamlining. Using *Prochlorococcus* MED4 as a model organism, we investigated the constraints on core genome stabilization using transcriptome profiling.

**Results:**

RNA-Seq technique was used to obtain the transcriptome map of *Prochlorococcus* MED4, including operons, untranslated regions, non-coding RNAs, and novel genes. Genome-wide expression profiles revealed that three factors contribute to core genome stabilization. First, a negative correlation between gene expression levels and protein evolutionary rates was observed. Highly expressed genes were overrepresented in the core genome but not in the flexible genome. Gene necessity was determined as a second powerful constraint on genome evolution through functional enrichment analysis. Third, quick mRNA turnover may increase corresponding proteins’ fidelity among genes that were abundantly expressed. Together, these factors influence core genome stabilization during MED4 genome evolution.

**Conclusions:**

Gene expression, gene necessity, and mRNA turnover contribute to core genome maintenance during cyanobacterium *Prochlorococcus* genus evolution.

## Background

The marine free-living cyanobacterium *Prochlorococcus* is the most abundant autotroph on our planet, yet its cell size and genome are nearly the smallest among the oxygenic phototrophs [1,2]. This bacterium geographically distributes throughout tropical and subtropical open seas, thriving particularly in oligotrophic regions [2,3]. The *Prochlorococcus* genus mainly consists of high-light (HL) and low-light (LL) ecotypes. These ecotypes display different vertical niche partitioning in water columns with stratified light and nutrient distributions [4].

Genome streamlining is an intriguing phenomenon that has long been observed in *Prochlorococcus* lineages [5]. Kettler *et al.* defined approximately 1250 genes as the core genome of *Prochlorococcus* based on a systemic analysis of 12 genome sequences of this clade, whereas more than 5000 genes were estimated within the flexible genome [6]. Although *Prochlorococcus* ecotype differentiation associated with flexible genome streamlining has been extensively studied [7-10], the mechanism in which the *Prochlorococcus* core genome is consistently maintained is unknown. It is hypothesized that core genes are more essential to a lineage than flexible genes [11,12], and thus, functional necessity dictates core genome stabilization. However, a growing body of evidences suggests that gene expression level is another important and independent predictor of molecular evolution from prokaryote to eukaryote [13-17]. Therefore, it is possible that *Prochlorococcus* genome stabilization and streamlining is not only influenced by functional gene necessity, and further transcriptome analyses are required to explain the genome evolution within this genus. Interestingly, the subspecies *Prochlorococcus* MED4 has an increased rate of protein evolution and a remarkably reduced genome [7,9,18]. These characteristics make it an ideal model organism for examining the evolutionary factors that influence genome evolution.

RNA-Seq is a high-throughput sequencing technique that has been widely used for transcriptome profiling [19,20]. It allows for the identification of operons, untranslated regions (UTRs), novel genes, and non-coding RNAs (ncRNAs) [21-24]. In order to determine the global features of MED4 transcriptome and provide insight for core genome stabilization at the angle of gene expression, we applied RNA-Seq to ten MED4 samples grown on Pro99 medium and artificial medium for *Prochlorococcus* (AMP) [25] and collected throughout its life cycle (Table 1; Methods). We identified the operon structure and UTRs, as well as novel opening reading frames (ORFs) and ncRNAs. By analyzing gene expression data, we infer that gene expression, gene necessity, and mRNA stability influence *Prochlorococcus* MED4 core genome stabilization.

**Table 1:** Summary of sequenced ten samples

**Sample**	**Total pair reads**	**Total mapped rate**	**Total mapped**	**Perfect mapped rate**	**Perfect mapped**	**Gene expression rate**		
						**All CDS genes**	**Core genome**	**Flexible genome**
esl1d	4,615,238	99.5%	4,590,777	97.4%	4,493,396	91.8%	95.1%	85.9%
esl3d	6,456,732	97.4%	6,288,857	90.9%	5,867,878	91.5%	94.7%	85.9%
esl4d	6,624,400	77.5%	5,133,248	75.8%	5,017,983	92.6%	95.9%	86.9%
esl8d	6,449,616	70.4%	4,540,530	70.0%	4,447,655	85.2%	89.0%	78.5%
esl10d	6,430,250	67.5%	4,337,847	64.6%	4,155,228	89.5%	93.0%	83.5%
amp3d	6,630,721	98.0%	6,499,433	93.6%	6,207,018	95.8%	98.2%	91.5%
s6_5h	6,401,265	88.2%	5,646,556	83.8%	5,361,059	88.5%	92.7%	81.1%
s6_10h	6,394,044	87.9%	5,617,168	83.4%	5,330,075	89.1%	93.1%	82.1%
s24_5h	6,391,818	84.8%	5,417,066	79.4%	5,075,743	92.9%	96.2%	87.0%
s24_10h	6,396,571	85.3%	5,453,077	79.2%	5,066,084	92.1%	95.3%	86.4%

esl1d: cultured one day in Pro 99; esl3d: cultured three days in Pro 99; esl4d: cultured four days in Pro 99; esl8d: cultured eight days in Pro 99; esl10d: cultured ten days in Pro 99; amp3d: cultured three days in AMP; s6_5h: Log-phase cells cultured five hours in AMP with 6 mM NaHCO_3_; s6_10h: Log-phase cells cultured ten hours in AMP with 6 mM NaHCO_3_; s24_5h: Log-phase cells cultured five hours in AMP with 24 mM NaHCO_3_; s24_10h: Log-phase cells cultured ten hours in AMP with 24 mM NaHCO_3_.

All cultures were incubated at 21°C and constantly irradiated with 28 μmol quanta m^-2^ s^-1^.

## Results

### Transcriptome structure of *Prochlorococcus* MED4

The Illumina high-throughput sequencing (RNA-Seq) protocols were applied to ten *Prochlorococcus* MED4 samples cultured in Pro99 and AMP (Table 1; Methods). Altogether, 62.8 million 90-bp pair-end reads were generated, and approximately 51.0 million pair-end reads (81.3%) were perfectly mapped to the genome (Table 1). Collectively, 91.8% of the MED4 genome was transcribed for at least one growth condition, and 61.2% of the genome was transcribed in all conditions. The transcribed regions might be larger if more growth conditions are tested. The genome expression cut-off was defined as the coverage of the tenth percentile of the lowest expressed genome regions [23] (Table 1). In contrast, 96.6% of 1965 coding-sequence (CDS) genes were expressed in at least one growth condition, and 80.9% were expressed in all conditions. Gene expression cut-off was defined as the mean RPKM (reads per kilobase per million mapped reads [26]) of the ten percentages of the lowest expressed gene regions (Table 1).

**Figure 1:**
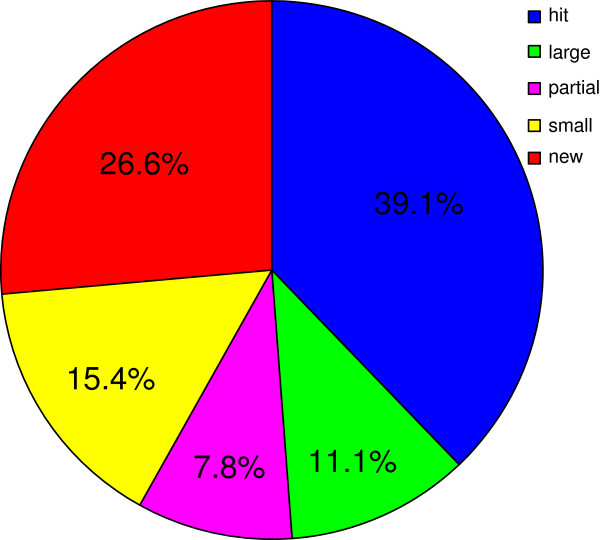
**Operon map comparison.** The operon map experimentally generated by this study compared with a bioinformatically predicted operon map generated by the Prokaryotic Operon DataBase (ProOpDB) [27]. “hit”: operons that were the same as predictions in ProOpDB; “large”: operons that contained more genes than predicted in the database; “partial”: operons that share partial genes with those in the database; “small”: operons that contain one or more genes predicted in the database; “new”: operons that were newly identified in this study.

The RNA-Seq reads mapping allow us to globally identify transcripts’ boundaries and adjacent gene regions [22-24]. To obtain a genome-wide operon map, a putative operon was characterized if it was repeatedly observed in at least three samples (Methods). Using this criterion, 55.5% of all genes were assigned to 422 primary operons (Additional file 1), representing the first operon map of *Prochlorococcus* based on experimental data. The operon map completely or partially shared 73.4% of operon genes within predicted operons identified by the Prokaryotic Operon DataBase [27]. The remaining operons comprised many new genes recently predicted by Kettler *et al.* and Steglich *et al.*[6,28] (Figure 1). The majority of the operons (63.0%) identified in this study were composed of two genes. The largest operon identified was a ribosomal protein operon containing 20 genes, and this was consistent with previously published observation made by Steglich *et al.*[29]. Furthermore, those extensively characterized operons, such as *kaiBC* circadian clock [30], two-component system *phoRB*[31], photosystem I core apparatus *psaAB*[32], and carboxysome shell proteins *cso* cluster [33], were also included in the operon map (Additional file 1).

UTRs were predicted by identifying the operons’ boundaries. These were defined as sharp declines in coverage of the regions upstream or downstream of the start or stop codons, respectively (Methods). Accordingly, 745 5’UTRs were identified and the median UTR length was approximately 29 nucleotides (nt) (Sheet 1 of Additional file 2). Although most 5’UTRs were small and typically similar to many other bacterial [24,34], 8.86% of the 5’UTRs identified were longer than 100 nt. Long 5’UTR, particularly in prokaryotes, may contain *cis*-regulation element(s) such as the Shine-Dalgarno (SD) sequence, which mediates mRNA translational efficiency. Potential RNA elements (5’UTR > 15 nt) were scanned using the Rfam [35], but no conserved elements were identified. These observations are in agreement with previous work [36] and suggest *Prochlorococcus* may contain unknown *cis*-regulatory sequences, like targets for ncRNAs.

We also identified 337 3’UTRs (Sheet 2 of Additional file 2). When these sequences (3’UTR > 10 nt) were searched by the ARNold [37], only 11 significant termination signals were identified (Sheet 2 of Additional file 2). However, the high proportion (35.6%) of long 3’UTRs (> 60 nt) suggests that these regions may have other important roles that require further exploration.

To identify new ORFs and ncRNAs, we analyzed the intergenic regions determined by current gene annotation (Sheet 2 of Additional file 3). Seven transcript units were identified with high confidence, including two ORFs and five ncRNAs (Additional file 4). The two ORFs were conserved hypothetical proteins present in related subspecies such as *P. marinus* MIT9202, *P. marinus* W9, and *P. marinus* MIT9515. All five identified ncRNAs were expressed in at least eight conditions (Additional file 4). In particular, TibYfr5 was the highest expressed ncRNA among five predicted ncRNAs, whereas TibYfr1 consistently showed the highest abundance under the light–dark conditions [38]. This suggests that TibYfr1 and TibYfr5 expression level may be influenced by changes in light.

### Highly expressed genes were overrepresented in the core genome but not in the flexible genome

**Figure 2:**
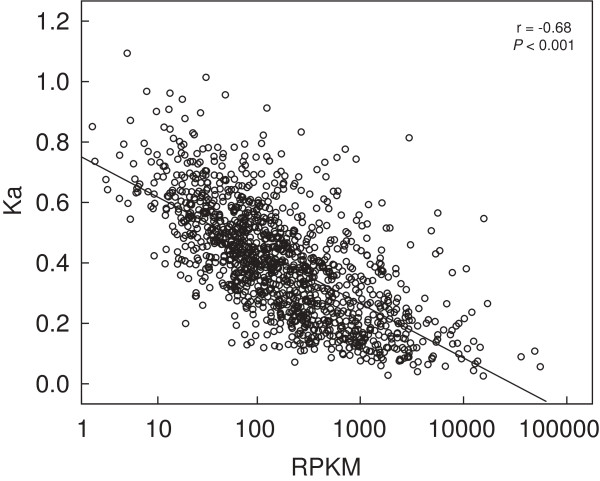
**Correlation between the gene expression levels and nonsynonymous substitution rates (Ka).** RPKM, reads per kilobase per million mapped reads; number of pairwise protein = 1275, Spearman’s r = -0.68, *P* < 0.001.

Using genome-wide expression data, we compared gene expression profiles between the MED4 core and flexible genomes [6]. Up to 94.3% of the 1251 genes in the core genome were expressed, and this was significantly higher than 84.9% of the genes expressed in the flexible genome (*P* < 0.001). Furthermore, a moderate but significant correlation was observed between the gene expression levels (mean RPKM of ten samples for each gene) and corresponding protein nonsynonymous substitution rates (Ka) (N = 1275, Spearman’s r = -0.68, *P* < 0.001; Figure 2). This observation that higher expressed genes evolve slowly, which has been observed in various organisms [13,15,17], might also be true in *Prochlorococcus* MED4.

To uncover the variations of gene expression and molecular conservation, all CDS genes were classified into five subclasses based on expression level. Briefly, first, we assumed that at a certain time point, some transcripts are highly expressed, and some are lowly expressed or not even transcribed. Then, excluding the non-expressed genes, we used quartation to classify all expressed genes to three expression level groups: the genes with the top 25% RPKM in a sample were defined as highly expressed genes (HEG), the lowest 25% were classified to lowly expressed genes (LEG), and the median group was defined as moderately expressed genes (MEG). Thus, if we trace one gene’s expression level across multiple samples, it might be constantly classified into HEG, MEG, LEG, or NEG (non expressed genes), which were collectively designated constantly expressed genes (CEG); otherwise, it was defined as variably expressed gene (VEG).

**Figure 3:**
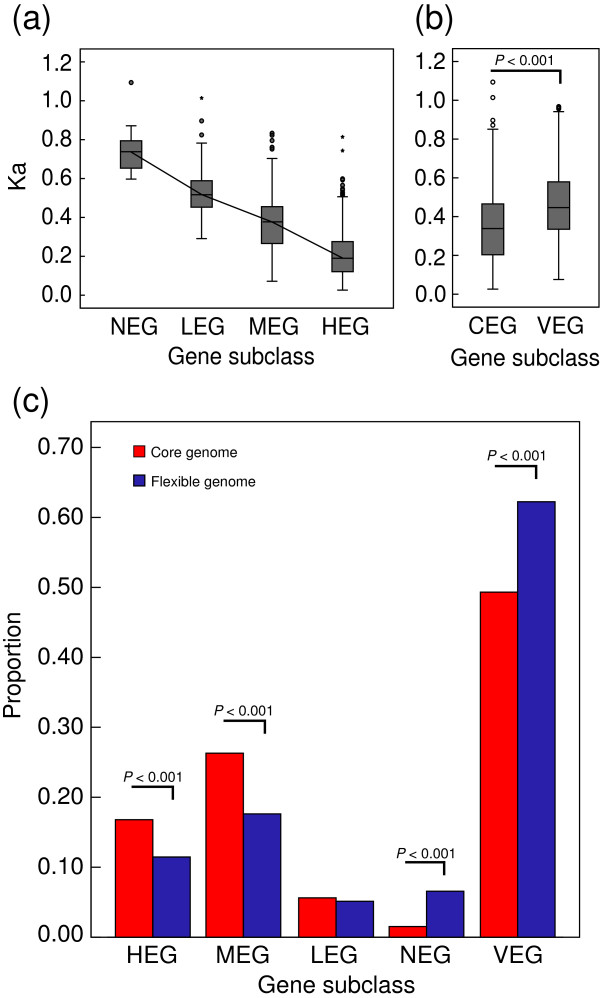
**Gene expression and molecular evolution of the core genome and flexible genome of *Prochlorococcus *MED4. (a)** Box plot of the correlation between gene expression levels and the nonsynonymous substitution rates (Ka). The line was drawn through the median. A circle represents an outlier, and an asterisk represents an extreme data point. **(b)** Nonsynonymous substitution rate comparison between CEG and VEG (Mann–Whitney U Test, two-tailed). A circle represents an outlier, and an asterisk represents an extreme data point. **(c)** Comparison of five expression subclasses between the core genome and flexible genome (Fisher’s exact test, one-tailed). *P*-value ≤ 0.05 was indicated in figure. HEG, highly expressed genes; MEG, moderately expressed genes; LEG, lowly expressed genes; NEG, non expressed genes; CEG, constantly expressed genes (including four expression subclasses mentioned above); VEG, variably expressed genes.

All MED4 CDS genes were classified into five subgroups (HEG, MEG, LEG, NEG, and VEG). HEG had a significantly lower nonsynonymous substitution rate (Ka) than MEG or LEG (Kruskal-Wallis Test, two-tailed *P* < 0.001; Figure 3a), indicating a strong negative correlation between gene expression level and evolutionary rate. Intriguingly, CEG subclass had a lower Ka than VEG (Mann–Whitney U Test, two-tailed *P* < 0.001; Figure 3b), even when HEG were excluded from the CEG because of their bias with the lowest evolutionary rate among all expression subclasses (data not shown).

Next, we compared the five gene expression subclasses of the core genome to that of the flexible genome. Our analysis clearly indicates that the genes in the HEG and MEG subclasses were more enriched in the core genome than in the flexible genome (17.7% > 11.5% and 26.8% > 15.3%, respectively; *P* < 0.001; Figure 3c). Conversely, the core genome had fewer NEG and VEG than the flexible genome (1.5% < 6.6% and 49.6% < 64.6%, respectively; *P* < 0.001; Figure 3c). These data strongly suggest that *Prochlorococcus* MED4 genes with constant high expression levels evolve slowly, and this concurs with previous findings in other prokaryotes and eukaryotes [13,15,17]. They also suggest that genes with relatively stable expression are more likely to evolve slowly when compared with VEG.

### Gene expression level and functional necessity independently influence core genome stabilization

**Figure 4:**
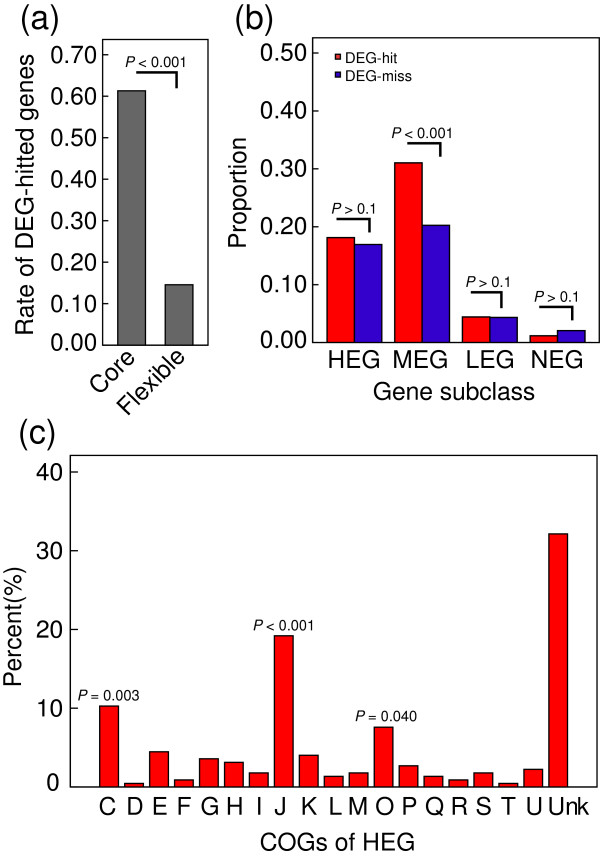
**Gene necessity analysis and COG functional enrichment of HEG.** All coding-sequence genes were searched on the Database of Essential Genes (DEG8.0 [39]) using BLASTx (E-value = 1 × 10^-4^). **(a)** Comparison of the DEG-hit genes in the core and flexible genomes. **(b)** Comparisons of gene expression subclasses between DEG-hit and DEG-miss genes. **(c)** COG functional enrichment of HEG in the core genome. Statistic significance was performed by Fisher’s exact test (one-tailed). *P*-value ≤ 0.05 was indicated in figure. COG, clusters of orthologous groups; Core, the core genome; DEG-hit, genes with homologs identified in the database; DEG-miss, genes without any known homologs; Flexible, the flexible genome; Unk, unknown function.

It is well established that the core genome contains more indispensible genes that play central metabolic roles [11,12]. This results in a lower mutation rate than in the flexible genome. To define essential genes, we searched for homologs of all MED4 coding genes in the Database of Essential Genes (DEG8.0), a database that collects all indispensible genes measured in laboratory by far [39]. Using BLASTx (E-value = 1 × 10^-4^), we found homologs for 871 MED4 coding genes. A total of 767 genes were distributed in the core genome, representing 61.3% of core genes. This was a significantly higher proportion of genes than those distributed in the flexible genome (14.6%; *P* < 0.001; Figure 4a). These data support the hypothesis that core genes are responsible for central cell metabolism in *Prochlorococcus*.

We also compared the expression levels of the core MED4 genes that had homologs in the DEG database (DEG-hit) with those genes that did not have any known homologs (DEG-miss). HEG, LEG, and NEG had no enrichment for either DEG-hit or DEG-miss genes (*P* > 0.1; Figure 4b). Although the MEG subclass had a significantly higher rate of DEG-hit genes (*P* < 0.001; Figure 4b), the mean expression level of the DEG-hit genes (mean RPKM = 602.62) was not significantly different from that of the DEG-miss genes (mean RPKM = 874.81; Student's t-test, two-tailed *P* = 0.084). Therefore, as previous works reported [14,40,41], this suggests that essential genes are not necessarily highly expressed and that gene expression levels relatively independently affect sequence evolution in *Prochlorococcus* MED4.

We also performed functional enrichment analysis on each gene expression subclass. As most of the genes in the flexible genome have no COG categories [42], we mainly focused on the core genes’ expression subclasses, especially the HEG. Among these core HEG genes, several functional categories were more prominent than others. These included the “C” (energy production and conversion), “J” (translation and ribosomal structure), and “O” (protein modification, folding and turnover) categories (Figure 4c). These results suggest that these central metabolic functions are among the most conserved throughout the evolution of *Prochlorococcus* lineage. In particular, translational and ribosomal components are generally regarded as the most stable part of genome [14,43]. In addition to ribosomal proteins, photosynthetic apparatus and energy metabolism genes were also overrepresented among the core genome. Interestingly, genes involved in protein modification and folding were also stably and highly expressed, suggesting that these genes are under strict constraints similar to those observed for ribosomal and photosynthetic genes.

Additionally, category “R” (general function) was slightly enriched in both LEG and NEG (*P* = 0.023 and 0.055; data not shown).

### Varied gene expression in different cellular processes

**Figure 5:**
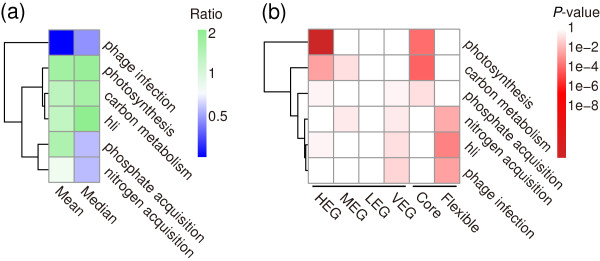
**Varied expression in six cellular processes, including photosynthesis**[45]**, carbon metabolism**[46]**, phosphate acquisition**[47]**, nitrogen acquisition**[46]**, *hli *(high-light inducible genes), and phage infection**[48]**. (a)** Expression profiles of six cellular processes. For each gene, the mean expression in ten samples was used as its expression value. For six functional categories, the mean and median expression values were normalized to values of ribosomal genes. **(b)** Enrichment analysis of four expression subclasses (HEG, MEG, LEG, and VEG) for six functional processes (Fisher’s exact test, one-tailed). Core: the core genome; Flexible: the flexible genome, HEG: highly expressed genes; MEG, moderately expressed genes; LEG, lowly expressed genes; VEG, variably expressed genes.

To investigate gene expression levels during different physiological processes, we compared the average gene expression levels of six important pathways using the ribosomal component as an expression standard because of its universally high expression level [14,44]. Six cellular pathways displayed significantly different expression levels (Kruskal-Wallis Test, two-tailed *P* < 0.001; Figure 5a). Photosynthesis and carbon metabolism pathway genes were expressed at the highest level (Figure 5a), and these data were consistent with HEG that function in energy production and conversion within the core genome (Figure 4). Subsequent enrichment analysis of the expression subclasses showed that HEG were overrepresented in both pathways (Figure 5b).

Intriguingly, *hli* genes exhibited high expression levels (Figure 5a). This may be due to the sustained light condition used in this study. However, HEG were not enriched among the *hli* genes (Figure 5b). We infer that is because several genes, such as *PMM1384* and *1385* (*hli12* and *hli11*), are highly expressed when cells are exposed to high light conditions (Sheet 3 of Additional file 3). On the contrary, *PMM1390* (*hli10*) was slightly transcribed (Sheet 3 of Additional file 3). It may that differentially expressed *hli* genes protect different cellular components, such as light harvesting antenna and nucleic acids [45,49].

As expected, phage-related genes displayed the lowest expression levels in this study, as phage infection conditions were not tested. It would be better to have phage infection condition data to analysis these genes expression profiles. For phosphorus and nitrogen acquisition genes, there was no significant enrichment in the four expression subclasses (Figure 5b). However, *PMM1119* and *PMM112* (two P-limitation-inducible porins) [47], and one ammonium transporter (*amt1*, *PMM0263*) were highly expressed (Sheet 3 of Additional file 3), suggesting that these proteins play particular roles in phosphorus or nitrogen uptake, respectively.

### Conserved genes more likely clustered to operon than poorly conserved genes

**Figure 6:**
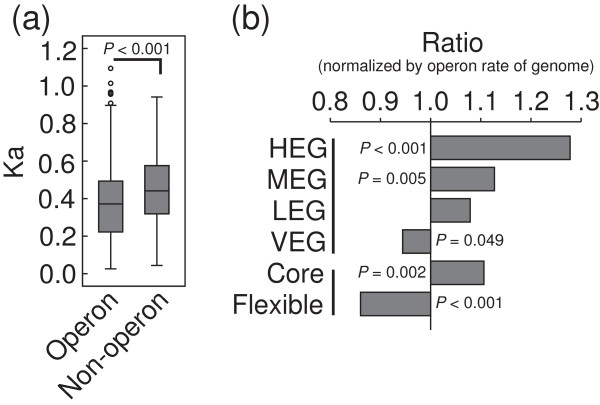
**Operon distribution of different expression subclasses. (a)** Comparison of nonsynonymous substitution rate between operon genes and non-operon genes in MED4 (Mann–Whitney U Test, two-tailed). A circle represents an outlier. **(b)** Operon rate of four expression subclasses (HEG, MEG, LEG, and VEG) or the core/flexible genomes (Fisher’s exact test, one-tailed). The operon rate was defined as the ratio of operon genes to total genes in a certain gene collection. The operon rate of each subclass was normalized by the whole genome operon rate (55.5%). *P*-value ≤ 0.05 was indicated in figure. Core, the core genome; Flexible, the flexible genome; HEG, highly expressed genes; MEG, moderately expressed genes; LEG, lowly expressed genes; VEG, variably expressed genes.

We identified 210 operons (49.8% of total) that uniquely belonged to the core genome, whereas the flexible genome harbored only 86 operons (20.4% of total). Based on this observation, we examined whether operon genes were more conserved than non-operon genes. The comparison of nonsynonymous substitution rates indicated that the total operon coding-sequence genes indeed evolve more slowly than non-operon genes (*P* < 0.001; Figure 6a). Furthermore, operon genes were significantly overrepresented in the core genome but not in the flexible genome (Figure 6b). Because HEG are more conserved in MED4, we compared the operon rate (the ratio of operon genes to total genes in a certain gene collection) of HEG with the other expression subclasses. We found that operons are strikingly enriched in HEG and MEG (Figure 6b). In addition, the distribution of operon size within the core genome when compared with the flexible genome was slightly different. Approximately 63.8% (134/210) of operons detected in the core genome harbored two genes, compared with 72.1% (62/86) in the flexible genome (*P* = 0.065). Extensive works has reported that essential genes prefer to be in operon [50,51]. We compared the operon rate of DEG-hit genes and DEG-miss genes. Significantly more operonic genes were indeed present in the former gene set (62.7% > 57.6%; *P* = 0.042). These findings strongly suggest that MED4 conserved genes are more likely to be co-transcribed and are larger in size.

### Constantly and abundantly expressed transcripts undergo quick degradation

**Figure 7:**
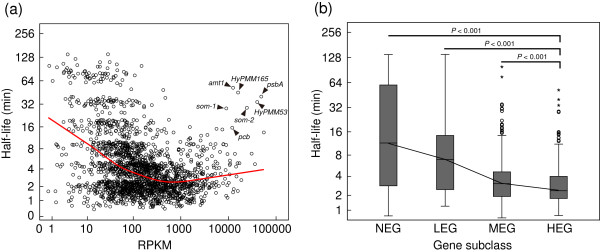
**Correlation between gene expression levels and mRNA half-lives. (a)** Correlation between gene expression levels and mRNA half-lives. Red line shows loess-smoothed curve. The exceptions reported by Steglich *et al.*[29] were indicated with arrows **(b)** Box plot of the correlation between gene expression levels and mRNA half-lives (Mann–Whitney U Test, two-tailed). The line was drawn through the median. A circle represents an outlier, and an asterisk represents an extreme data point.

Pál *et al*. previously reported a weak positive association between the rate of evolution and mRNA half-lives in yeast [13]. However, the analysis was done by incomplete genome dataset of RNA degradation. Using a genome-wide mRNA half-life dataset [29], we observed a similar but also slight tendency for genes with lower Ka to have shorter half-lives (N = 1262, Spearman’s r = 0.29, *P* < 0.001). Further investigation showed that highly expressed genes were more likely degraded fast (Figure 7a). Intriguingly, as Steglich *et al.* reported [29], several genes, including *amt1* (ammonium transporter, *PMM0263*), *psbA* (PsbA protein D1, *PMM0223*), *som-1/2* (porins, *PMM1119* and *PMM1121*), *pcb* (light harvesting complex protein, *PMM0627*), and also two hypothetical genes (*HyPMM53* and *HyPMM165*), that were strongly transcribed turnover very slowly (Figure 7a). This may attribute to these genes’ specific roles in these growth conditions. Despite these exceptions, similar result indicated that highly expressed transcripts had significant shorter half-lives (Kruskal-Wallis Test, two-tailed *P* < 0.001; Figure 7b). Accordingly, the mRNA turnover rate for genes within the core genome was faster than that of the flexible genome (*P* < 0.001). Besides for the advantages of rapid recycling nucleotides to adapt to oligotrophic environment [29], fast turnover of HEG might also be beneficial for translation fidelity [52], and consequently make the core genome more economical and compatible with cellular physiology.

## Discussion

*Prochlorococcus* is a typical phototroph whose cellular physiology and transcriptome are comprehensively affected by photoperiod [38,46]. We wondered whether light cycle-influenced gene expression profiles might lead to contradictory conclusions regarding the correlation between gene expression and evolution traits when *Prochlorococcus* is cultured under constant light conditions. Therefore, we applied the same method we developed to light–dark expression data generated by RNA-Seq [38]. First, we again observed a significant correlation between gene expression levels and corresponding nonsynonymous substitution rates (N = 1275, Spearman’s r = -0.69, *P* < 0.001; Additional file 5), and this was confirmed by the comparisons of evolution rates of four expression subclasses (*P* < 0.001; Additional file 6a). Second, constantly expressed genes, particularly HEG and MEG with lower Ka, were most often located within the core genome (Additional file 6c). Third, lowly expressed genes were more likely slowly degraded (Additional file 7a), and four of seven exceptions described above (Figure 7a) retained in this light–dark conditions (Additional file 7a). The comparisons of gene expression subclasses further indicated constantly and highly expressed transcripts tend to be quickly degraded (Additional file 7b). Interestingly, there was no significant difference between HEG and MEG (*P* > 0.1, Additional file 7b), and the same trait was also observed in the correlation between gene expression levels and half-lives when expression level increased to a certain degree the decay rate no longer declined (Figure 7a and Additional file 7a). These observations might be partially caused by specific growth conditions, or alternatively, by the genes’ position in operon because those genes located at 3’-end of operons are less expressed but slower degraded than 5’-end genes [29]. Therefore, half-lives of the high-operon-rate genes, such as HEG and MEG (Figure 6b), are more likely dependent upon their positions in operons. Despite opronic genes’ position, degradation distinction still can be observed in those genes with great difference in expression levels (like HEG versus LEG). However, it is not simplistic to figure out what extent the gene position can influence half-life to, and this also deviates from our topic in this study.

Although all experimental conditions tested in this study are considered physiologically normal, we also wonder whether environmental stress, such as iron that was studied by Thompson and coworkers [53], may affect the correlation between gene expression levels and molecular evolution. First, similar results were observed that highly and constantly expressed genes had lower Ka (Additional file 8a and b), and they were enriched more within the core genome (Additional file 8c). Second, those genes with constantly high expression level (HEG and MEG) had short half-lives (Additional file 9). Nonetheless, all of our observations are in accordance with previous conclusions drawn from normal growth conditions under constant illumination, and this may indicate that gene expression levels have relatively self-contained influence on genome evolution in *Prochlorococcus* MED4. But note that the conditions we have tested are actually in the laboratory, the similar study conducted using the cultures *in situ* will facilitate to further elucidate the core genome stabilization of *Prochlorococcus*.

Genes within the flexible genome are subject to relaxed constraints, and these genes can undergo frequent gain and loss in *Prochlorococcus*, leading to isolates differentiation. Multiple factors such as an incomplete DNA-repair system, relaxed selection, and paralog deletion can contribute to genome reduction [7-10]. However, little is known about factors that affect the molecular evolution of the *Prochlorococcus* core genome. Gene expression level has been reported as an independent factor that influences the rate of protein evolution across taxa [13,14,17,54]. In this study, we have provided evidences that highly conserved genes were more likely to be abundantly expressed, and highly and constantly expressed genes were distributed more in the core genome than in the flexible genome (Figures 2 and 3). Selection pressure imposes on those highly expressed genes to minimize the great cost (or toxicity) of corresponding mistranslated or error-folded proteins [17,55]. As the core genes show higher expression levels, these genes accordingly undergo more powerful evolutionary constraints derived from translation and folding [17]. Because efficient and fast mRNA degradation can minimize the use of poor mRNA and thus reduce the production of low-quality polypeptides derived from translation errors [52], highly expressed genes are more likely to be quickly degraded. This in turn increases the cellular fitness of abundantly expressed core genes. Notably, genes involved in protein folding and turnover were stably and highly expressed (Figure 4c). This has also been observed in natural microbial communities revealed by metatranscriptomic data [56]. These findings suggest that *Prochlorococcus* invests in protein folding and degradation to ensure protein fidelity, and thus further increases translational robustness.

However, it is reasonable to assume that essential genes are more likely abundantly expressed, thus the core genome that is of high necessity has higher expression level. Previous reports have demonstrated the difficulties in accepting this assumption [14,40]. Our result also suggests that expression level is relatively independent of gene necessity in *Prochlorococcus* MED4, as no significant difference in gene expression levels was observed between genes with conserved essential homologs (DEG-hit) and those without homologs (DEG-miss) (Figure 4b). In terms of which one contributing more than the other, the better model is required in the future.

The gene necessity (or indispensability) [57] influences the core genome stabilization because of its essential functions for physiology and metabolism. In particular, we found that energy metabolism, protein synthesis, and protein folding genes were more enriched in HEG within the core genome (Figure 4c). This implies that these central metabolic pathways lie in the most conserved gene pool across the evolutionary history of *Prochlorococcus*. Therefore, by analyzing mRNA levels, we were able to reach the same conclusion as those drawn by comparative genomics and protein sequence alignments [43]. Additionally, operons were more likely distributed in the core genome than in the flexible genome (Figure 6b). Basically, this distinction might be derived from the higher proportion of essential genes in the core genome (Figure 4a). Important genes more likely cluster to operons because those central metabolic genes, such as photosynthetic apparatus or ribosome machinery, in the same operon can be beneficially co-regulated and co-transcribed, and (or) packed to a complex [50,51,58].

## Conclusions

We used RNA-Seq to obtain a blueprint of the transcriptome of *Prochlorococcus* MED4. We identified remarkable distinctions in gene expression levels, gene necessity, and mRNA turnover between the core and flexible genomes, indicating that they are powerful constraints imposed on core genome stabilization. We hope these findings will contribute to a better understanding of the causes of ecotypic differentiation in the *Prochlorococcus* genus, and offer a new perspective for future investigations of cyanobacterium evolution.

## Methods

### Growth of *Prochlorococcus* MED4

*Prochlorococcus* MED4 strains were cultured in Pro99 medium and AMP [25] at 21°C with an irradiance of 28 μmol quanta m^-2^ s^-1^. Before the experiment, the cultures were maintained under continuous light at the stationary phase for five generations. Then 8 ml of stationary-phase cell cultures were inoculated into 92 ml of indicated growth medium (Table 1). For the Pro99, cells were harvested throughout the life cycle. These included lag-phase (esl1d), early log-phase (esl3d), middle log-phase (esl4d), stationary phase (esl8d), and post-stationary phase (esl10d) (Additional file 10). For AMP, stationary-phase cells were grown with varying concentrations of sodium bicarbonate (0 mM, 6 mM, and 24 mM) [25] for two time periods (5 hours, 10 hours; Table 1) (our primary aim was to maximize the number of transcripts represented under normal growth conditions). Each growth condition was performed in triplicate. Chlorophyll fluorescence was monitored on a Plate reader (Spectra Max M2^e^, Molecular Devices), with an excitation wavelength of 440 nm and an emission wavelength of 680 nm.

### Total mRNA preparation

To extract total mRNA, one volume of each culture was fixed with three volumes of RNA-later (15 mM EDTA, 18.75 mM sodium citrate, and 525 g/l ammonium sulfate), harvested by filtration (0.22 μm cellulose membrane), snap frozen in liquid nitrogen, and stored at -80°C. Before RNA extraction, cells were treated with 150 ml 10 mM Tris–HCl (pH 7.5), 2 ml RNase inhibitor (20 U/μl, AM2696), and 1 ml Readylysis lysozyme (Epicentre). Total RNA was extracted using the mirVana RNA isolation kit according to the manufacturer’s instructions (Ambion). DNA was removed by using Turbo DNA-free™ Kit (Ambion). Quality of the total RNA samples was assessed using the Nanodrop spectrophotometer (Thermo) and agarose gel electrophoresis. The total RNA of each triplicate culture was extracted separately, and mixed together (~8 μg) after measuring the quality of each sample.

### cDNA synthesis, DNA sequencing and reads mapping

cDNA synthesis was performed using the standard protocol of Shenzhen BGI (China) [59]. Briefly, the rRNA-depleted mRNA (for details see BGI patent WO2012083832 A1) was fragmented and then random primers were used to synthesize the first-strand cDNA. The second-strand cDNA was synthesized with DNA polymerase I. Short fragments were purified with QiaQuick PCR extraction kit (Qiagen), and then were sequenced under the Illumina HiSeq™ 2000 platform at Shenzhen BGI. The full sequencing technical details can be inspected in the services of BGI (http://www.genomics.cn). This yielded approximately six million 90-bp pair-end reads for each sample (Table 1). Then pair-end reads were mapped to the *Prochlorococcus* MED4 genome (accession number: NC_005072) using Bowtie2 [60] with at most one mismatch. The coverage of each nucleotide was calculated by counting the number of reads mapped at corresponding nucleotide positions in the genome. The number of reads that were perfectly mapped to a gene region was calculated using BEDTools [61], and then it was normalized by gene length and total mapped reads, namely RPKM as the gene expression value [26]. The gene annotations for *Prochlorococcus* MED4 were downloaded from MicrobesOnline [62] with modifications for non-annotated genes that were designated “HyPMM#”. New ORFs identified in this study were annotated with “TibPMM#” (Sheet 2 of Additional file 3). Sequences generated by this study are available in the Gene Expression Omnibus (GEO) under accession number GSE49517.

### Identification of operons and UTRs

Using a *priori* knowledge of the translation start and stop site from Additional file 3, the coverage of ORF upstream and downstream regions was scanned to identify a point of sharp coverage decline. To define the boundary, we applied criteria modified from Vijayan *et al.*[24]. Briefly, a transcript’s boundary (translation start or stop site was defined as *i* = 0, and “*i + 1*” is the upstream or downstream of position “*i*”) was defined when position “*i*” satisfied one of the following three criteria: (1) coverage(*i*)/coverage(*i + 1*) ≥ 2, binomial_cdf_ (coverage(*i + 1*), coverage(*i*) + coverage(*i + 1*), 0.5) < 0.01 and coverage(*i + 1*) > coverage(*i*:(*i-89*))/(90 × 7); (2) coverage(*i*)/coverage(*i + 1*) ≥ 5 or coverage(*i*)/coverage(*i + 2*) ≥ 5, and coverage(*i + 1*) < coverage(*i*:(*i-89*))/(90 × 7); (3) coverage(*i + 1*) ≤ background. Where binomial_cdf_ (*x*, *n*, *p*) is the probability of observing up to *x* successes in *n* independent trials when success probability for each trial is *p*. We assumed reads were uniformly distributed on position “*i*” and “*i + 1*” (*p* = 0.5). If a sharp coverage reduction occurred, coverage(*i + 1*) would be much smaller than coverage(*i*); that was, the success of coverage(*i + 1*) became a small probability event in the events of all reads mapped to “*i*” and “*i + 1*” (binomial_cdf_ < 0.01). The strictest criterion (1) was used for highly transcribed genes. Since the coverage of a certain transcript is uneven from the 5’-end to the 3’-end for sequencing bias [63], we checked the coverage of each gene’s left and right 90-bp nucleotides to define whether the gene’s upstream or downstream regions were transcribed at high or low levels. For position “*i*”, if its coverage was higher than 1/7th of the mean coverage of the upstream or downstream 90-bp (Sheet 1 of Additional file 3), this position would be examined by criterion (1) for the boundary definition. Otherwise, it fell under criterion (2). If the reduction of coverage was not sufficient for the above two criteria, the boundary would be defined by genome background (Sheet 1 of Additional file 3), which was determined as the tenth percentile of the lowest expressed nucleotides within gene regions [23].

The 5’UTR was defined as the upstream sequence from the translation start site of transcript, and 3’UTR was the downstream sequence from the translation stop site. If the adjacency of two ORFs located on the same strand had no sharp coverage reduction that was filtered by the three criteria described above, two ORFs belonged to a single operon. To obtain a robust operon map, operons that were repeatedly observed in at least three samples were considered reliable. The operon map was manually proofread to account for unpredictable fluctuations in computing.

### Novel gene identification

The intergenic regions were scanned to identify new genes. A rapid coverage reduction was considered the end of the new transcript, and this was confirmed by manual assessment. Putative transcripts were analyzed using BLASTn (E-value = 1 × 10^-3^, word = 4) and BLASTp (E-value = 1 × 10^-4^, word = 3) to confirm homologs of these putative proteins. Next, candidate ORFs were predicted by GeneMark [64] using *Prochlorococcus* MED4 as the training model. The remaining transcripts that were filtered by BLAST were defined as putative ncRNAs.

### Enrichment analysis

Enrichment analysis involves the statistically identification of a particular function category or expression subclass that is overrepresented in the whole gene collection. Since many cases in our study contained a small number of genes, we used Fisher’s exact test (one-tailed) for enrichment analysis (Fisher’s exact test were applied for all statistic significance tests in this study unless otherwise indicated). Some genes without COG were not excluded so the enrichment was fully representative. COG functional groups can be inspected in COGs database [42].

### Estimating synonymous (Ks) and nonsynonymous (Ka) substitution rate

The complete genome sequences of *Prochlorococcus* SS120, *Prochlorococcus* MIT9313, and *Synechococcus* CC9311 (accession number: NC_005042, NC_005071, and NC_008319) were downloaded from NCBI. Annotations were obtained from Kettler *et al.*[6]. Pairwise calculations of Ka and Ks of *Prochlorococcus* MED4 orthologs compared with each of the three related species were performed using software YN00 in the package PAML [65]. To analyze the correlation between Ka and gene expression levels, mean Ka values of the three ortholog pairs were used.

## Abbreviations

AMP: Artificial medium for *Prochlorococcus*; bp: Base pairs; CDS: Coding DNA sequence; CEG: Constantly expressed genes; COG: Clusters of orthologous groups [42]; DEG: Database of essential genes [39]; HEG: Highly expressed genes; Ka: Nonsynonymous substitution rate; Ks: Synonymous substitution rate; LEG: Lowly expressed genes; MEG: Moderately expressed genes; NCBI: National Center for Biotechnology Information; ncRNA: Non-coding RNA; NEG: Non expressed genes; nt: nucleotides; ORF: Open reading frame; RPKM: Reads per kilobase per million mapped reads; SD: Shine-Dalgarno sequence; UTR: Untranslated region; VEG: Variably expressed genes; Yfr: Cyanobacterial functional RNA.

## Competing interests

The authors declare that they have no competing interests.

## Authors’ contributions

BW, CT, and YM conceived and designed the project. BW and CT analyzed the data and wrote the paper. LL and HL performed the cultures materials preparation. HJ and GQ participated in bioinformatics analysis. All authors have read and approved the final manuscript.

## Supplementary Material

Additional file 1 **Operons (harboring at least two genes) of *Prochlorococcus *** **MED4.** Click here for file

Additional file 2 **UTRs of *Prochlorococcus *** **MED4.** Sheet 1: 5’UTRs; sheet 2: 3’UTRs. Click here for file

Additional file 3 **RNA sequencing profiles and gene expression.** Sheet 1: summary of RNA-Seq for ten samples; sheet 2: gene annotations from MicrobesOnline [63] and expression classification; sheet 3: expression values of the whole genome. Click here for file

Additional file 4 Novel ORFs and ncRNAs. Click here for file

Additional file 5 **Correlation between the gene expression levels and nonsynonymous substitution rates (Ka) based on light–dark RNA-Seq data**[38]**.** RPKM, reads per kilobase per million mapped reads; number of pairwise protein = 1275, Spearman’s r = -0.69, *P* < 0.001. Click here for file

Additional file 6 **Gene expression and molecular evolution of the core genome and flexible genome of *Prochlorococcus *** **MED4 based on light–dark RNA-Seq data**[38]**. (a)** Box plot of the correlation between gene expression levels and the nonsynonymous substitution rates (Ka). The line was drawn through the median. A circle represents an outlier, and an asterisk represents an extreme data point. **(b)** Nonsynonymous substitution rate comparison between CEG and VEG (Mann–Whitney U Test, two-tailed). A circle represents an outlier, and an asterisk represents an extreme data point. **(c)** Comparisons of five expression subclasses between the core genome and flexible genome (Fisher’s exact test, one-tailed). *P*-value ≤ 0.05 was indicated in figure. HEG, highly expressed genes; MEG, moderately expressed genes; LEG, lowly expressed genes; NEG, non expressed genes; CEG, constantly expressed genes (including four expression subclasses mentioned above); VEG, variably expressed genes. Click here for file

Additional file 7 **Correlation between gene expression levels and mRNA half-lives based on light–dark RNA-Seq data**[38]. **(a)** Correlation between gene expression levels and mRNA half-lives. Red line shows loess-smoothed curve. The exceptions reported by Steglich *et al.* were indicated with arrows. **(b)** Box plot of the correlation between gene expression levels and mRNA half-lives (Mann–Whitney U Test, two-tailed). The line was drawn through the median. A circle represents an outlier, and an asterisk represents an extreme data point. Click here for file

Additional file 8 **Gene expression and molecular evolution of the core genome and flexible genome of *Prochlorococcus *** **MED4 based on iron-stress microarray data**[53]. **(a)** Box plot of the correlation between gene expression levels and the nonsynonymous substitution rates (Ka). Because microarray data quantify the relative expression level, no genes were classified to the NEG. The line was drawn through the median. A circle represents an outlier, and an asterisk represents an extreme data point. **(b)** Nonsynonymous substitution rate comparison between CEG and VEG (Mann–Whitney U Test, two-tailed). A circle represents an outlier, and an asterisk represents an extreme data point. **(c)** Comparisons of five expression subclasses between the core genome and flexible genome (Fisher’s exact test, one-tailed). *P*-value ≤ 0.05 was indicated in figure. HEG, highly expressed genes; MEG, moderately expressed genes; LEG, lowly expressed genes; CEG, constantly expressed genes (including three expression subclasses mentioned above); VEG, variably expressed genes. Click here for file

Additional file 9 **Correlation between gene expression levels and mRNA half-lives based on iron-stress microarray data**[53]**.** Box plot of the correlation between gene expression levels and mRNA half-lives (Mann–Whitney U Test, two-tailed). The line was drawn through the median. A circle represents an outlier, and an asterisk represents an extreme data point. Click here for file

Additional file 10 **Representative growth curve of *Prochlorococcus *** **MED4 in Pro99 medium.** The RNA collection points were indicated with arrows. The stationary-phase cells (esl8d) were inoculated into indicated medium for growth (Methods). Click here for file
