# Supplementary figures and images for: The transcriptome landscape of Prochlorococcus MED4 and the factors for stabilizing the core genome
Source: BMC Microbiol. 2014 Jan 18;14:11. doi: 10.1186/1471-2180-14-11 (PMC3898218; doi:10.1186/1471-2180-14-11)

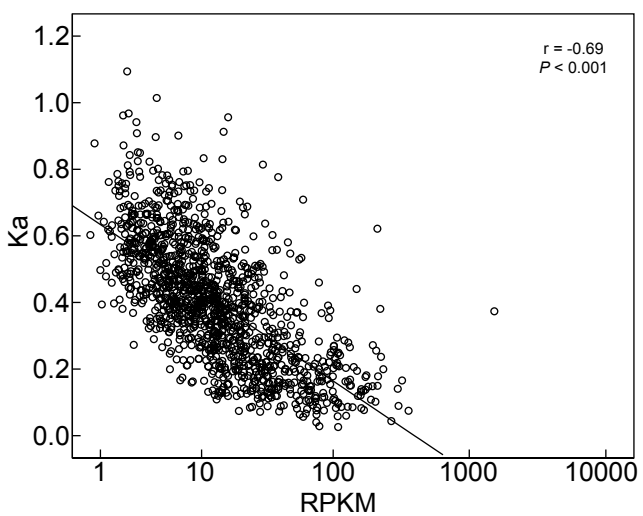

Supplement: Additional file 5 — Correlation between the gene expression levels and nonsynonymous substitution rates (Ka) based on light–dark RNA-Seq data[38]. RPKM, reads per kilobase per million mapped reads; number of pairwise protein = 1275, Spearman’s r = -0.69, P < 0.001. [file 1471-2180-14-11-S5.pdf]

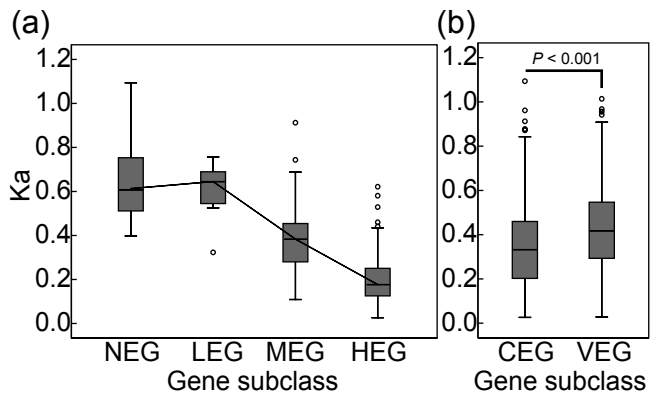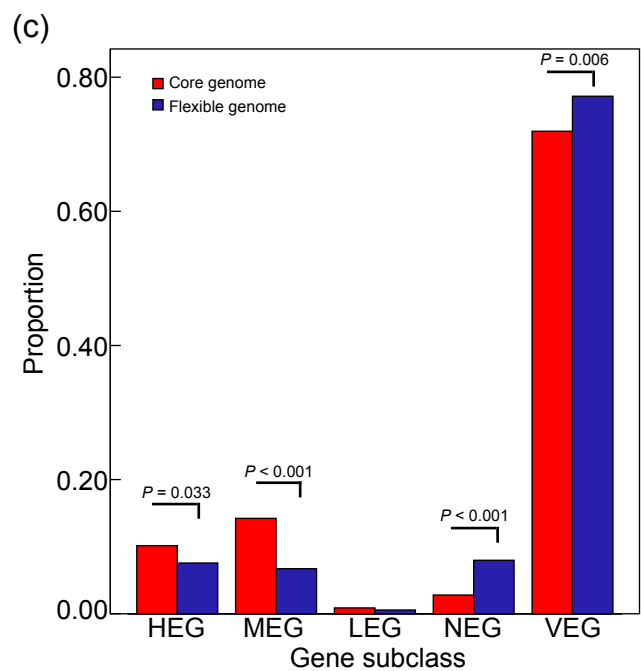

Supplement: Additional file 6 — Gene expression and molecular evolution of the core genome and flexible genome of Prochlorococcus MED4 based on light–dark RNA-Seq data[38]. (a) Box plot of the correlation between gene expression levels and the nonsynonymous substitution rates (Ka). The line was drawn through the median. A circle represents an outlier, and an asterisk represents an extreme data point. (b) Nonsynonymous substitution rate comparison between CEG and VEG (Mann–Whitney U Test, two-tailed). A circle represents an outlier, and an asterisk represents an extreme data point. (c) Comparisons of five expression subclasses between the core genome and flexible genome (Fisher’s exact test, one-tailed). P-value ≤ 0.05 was indicated in figure. HEG, highly expressed genes; MEG, moderately expressed genes; LEG, lowly expressed genes; NEG, non expressed genes; CEG, constantly expressed genes (including four expression subclasses mentioned above); VEG, variably expressed genes. [file 1471-2180-14-11-S6.pdf]

(a)

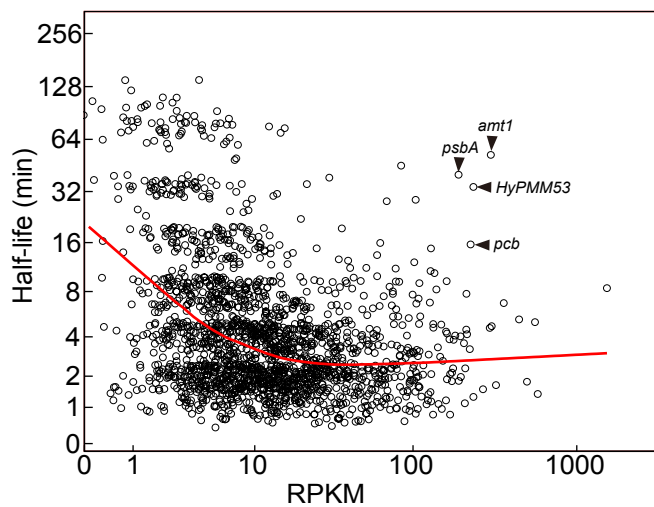

(b)

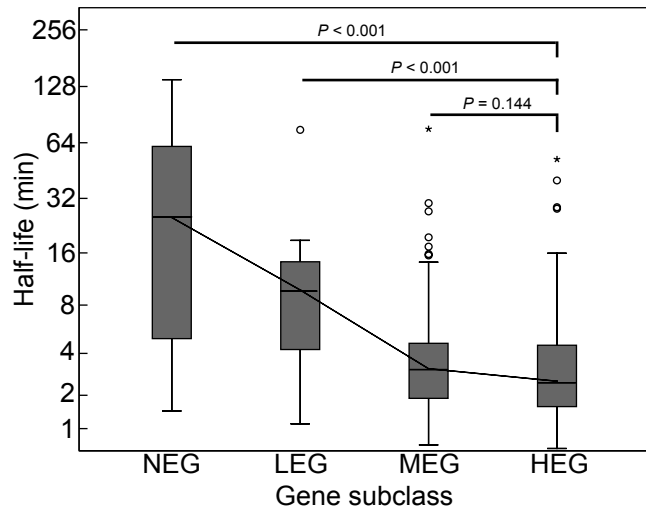

Supplement: Additional file 7 — Correlation between gene expression levels and mRNA half-lives based on light–dark RNA-Seq data[38]. (a) Correlation between gene expression levels and mRNA half-lives. Red line shows loess-smoothed curve. The exceptions reported by Steglich et al. were indicated with arrows. (b) Box plot of the correlation between gene expression levels and mRNA half-lives (Mann–Whitney U Test, two-tailed). The line was drawn through the median. A circle represents an outlier, and an asterisk represents an extreme data point. [file 1471-2180-14-11-S7.pdf]

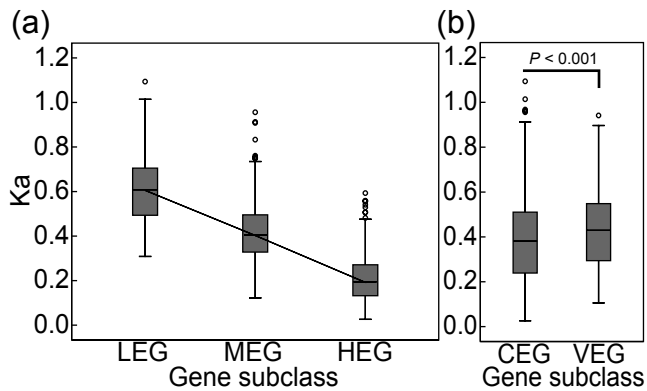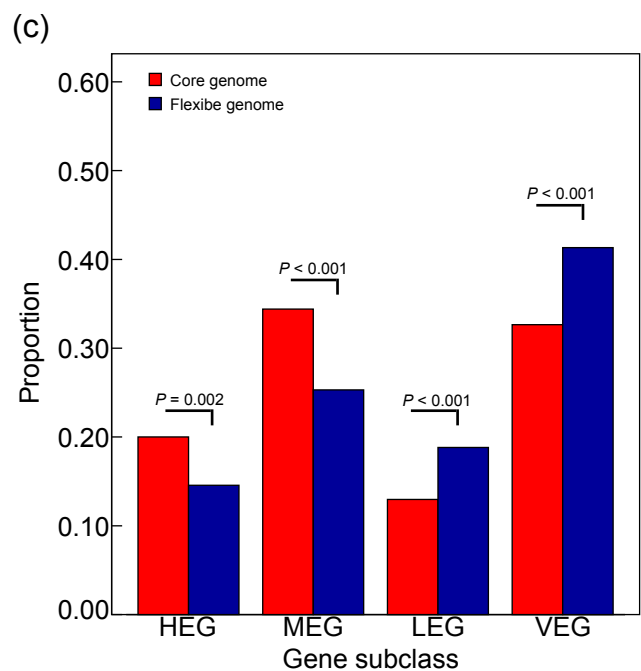

Supplement: Additional file 8 — Gene expression and molecular evolution of the core genome and flexible genome of Prochlorococcus MED4 based on iron-stress microarray data[53]. (a) Box plot of the correlation between gene expression levels and the nonsynonymous substitution rates (Ka). Because microarray data quantify the relative expression level, no genes were classified to the NEG. The line was drawn through the median. A circle represents an outlier, and an asterisk represents an extreme data point. (b) Nonsynonymous substitution rate comparison between CEG and VEG (Mann–Whitney U Test, two-tailed). A circle represents an outlier, and an asterisk represents an extreme data point. (c) Comparisons of five expression subclasses between the core genome and flexible genome (Fisher’s exact test, one-tailed). P-value ≤ 0.05 was indicated in figure. HEG, highly expressed genes; MEG, moderately expressed genes; LEG, lowly expressed genes; CEG, constantly expressed genes (including three expression subclasses mentioned above); VEG, variably expressed genes. [file 1471-2180-14-11-S8.pdf]

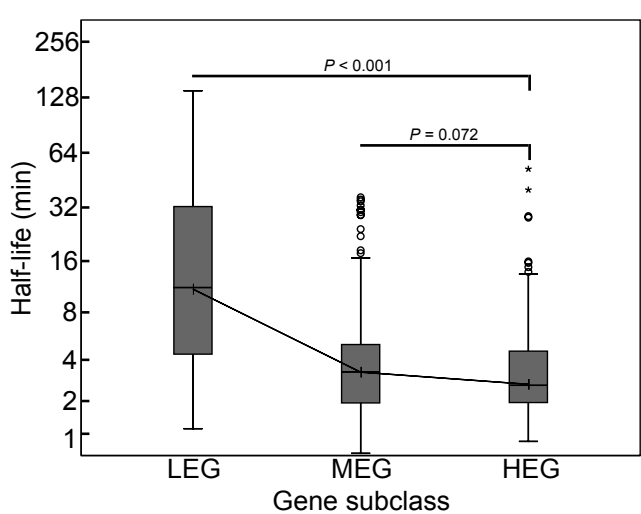

Supplement: Additional file 9 — Correlation between gene expression levels and mRNA half-lives based on iron-stress microarray data[53]. Box plot of the correlation between gene expression levels and mRNA half-lives (Mann–Whitney U Test, two-tailed). The line was drawn through the median. A circle represents an outlier, and an asterisk represents an extreme data point. [file 1471-2180-14-11-S9.pdf]

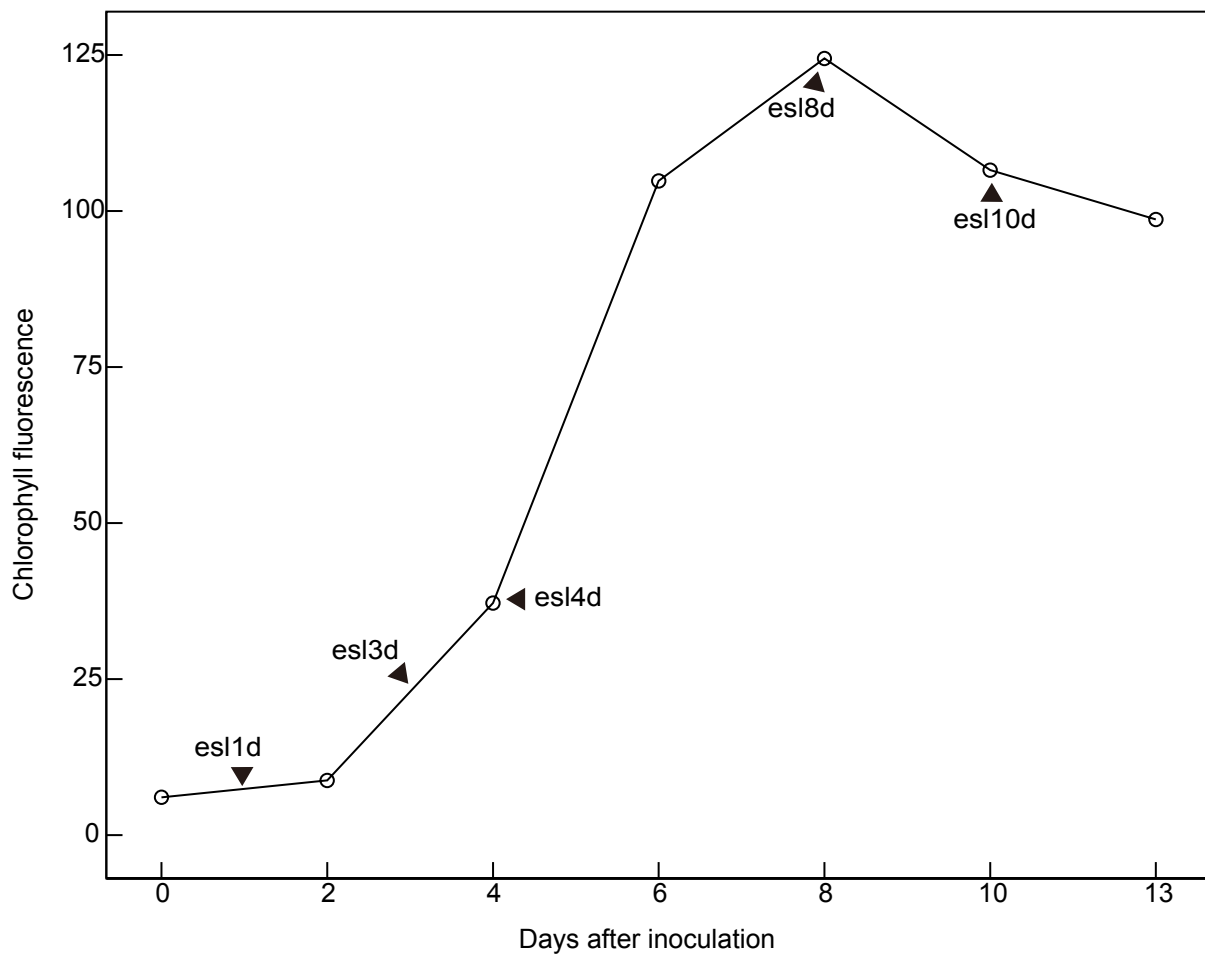

Supplement: Additional file 10 — Representative growth curve of Prochlorococcus MED4 in Pro99 medium. The RNA collection points were indicated with arrows. The stationary-phase cells (esl8d) were inoculated into indicated medium for growth (Methods). [file 1471-2180-14-11-S10.pdf]
